# Lunacy in Scotland

**Published:** 1898-10-01

**Authors:** 


### The Institutional Workshop.

### LUNACY IN SCOTLAND.

The Commissioners in Lunacy for Scotland present their fortieth annual report to Lord Balfour of Burleigh, Her Majesty's Secretary for Scotland, and, considering that the volume is necessarily in great part made up of statistics and details, it may be said to form unusually interesting reading for that class of literature. It is unquestionable that the Scottish Commissioners place their facts before us in a more taking way than do the English Commissioners, and we also notice that there is an almost total absence of that conflict between the Commissioners and the asylum authorities which is far from unknown in England. The causes for the satisfactory state of things existing in Scotland may be that the Visiting Commissioners are all medical men, that they visit singly, and that their reports are not presented until they have been shown to the asylum authorities.

The total number of insane people in Scotland, that is, the total number officially known, was 14,906 at the end of the year 1897, and of this total 2,295 were private patients, and 56 were State patients. It would seem that the number of lunatics in Scotland increased by 406 during the year 1897; and that 73 were private patients and 333 were paupers. Not the least gratifying fact in the statistics is that all the paupers were in public institutions, a state of things which once again compares favourably with England, where large numbers of patients are farmed out at an enormous extra cost to the ratepayers.

The Commissioners do not give us a decided opinion whether insanity is or is not increasing in Scotland at a higher rate than is accounted for by the increase of population; but they point out that in the more northern counties there has been an actual decrease of insanity compared with 1896, and that the central counties have increased the number of the insane, and that the chief offender in this respect has been Lanarkshire, which contributes not fewer than 199 of the 406; but this great increase is, no doubt, to some extent explained by the fact that Lanarkshire was for some time short of asylum accommodation, and that the increase followed sharply on the opening cf three new asylums in the county. No fact connected with asylum statistics has been oftener exemplified than the one that the certified insane increase in proportion to the provision made for them. Nevertheless, should the lunatic population continue to go upwards in Lanarkshire and downwards in Ross-shire, we may one day see the sane people migrate in a northerly direction, a condition which historic and even prehistoric footprints show never before existed.

There were scattered throughout the asylums during the year 67 voluntary patients. These are not under certificates—enter the asylum at their own wish, and may leave any time on three days' notice.

Of the asylums more particularly referred to, we notice that the Edinburgh Royal is spoken of as having fully developed its hospital character, and that the Glasgow Royal does its charitable work well.

The Commissioners reiterate their opinion—and we have pleasure in quoting it—that they "do not regard it as desirable that any class of persons should be brought under official supervision, unless such supervision appears to be necessary to guard against abuse." By taking this enlightened view the Commissioners may feel assured that they are contributing not a little to the comforts of many patients, and that the stigma of insanity is often averted from the individual and from the family.

It would seem from a sentence in the Blue Book that the District Boards have not the power to build for the class of patients between the pauper and the lower section of the middle class. Here is a real grievance, and it is to be desired that these District Boards will as soon as possible have conferred on them the same powers as have the English County Councils, for assuredly there is no class of the insane so much to be pitied as that which cannot be, and ought not to be, grouped with the paupers, and yet fails to command sufficient funds to be admitted to an ordinary private asylum.

The expenditure in 1896 amounted to £25 3s. 4d. per annum for each lunatic confined in a public establishment, and during last year there was a slight fall in this; but when an average is taken of all the paupers in asylums, in poorhouses, and in private dwellings, it is seen that there was a slight rise from £23 9s. 3d. to £23 11s.

Under the heading dealing with dangerous lunatics we learn that only 13 were sent to asylums under the section of the Act which applies to such cases. This seems an extraordinarily small number, and the explanation given is that proceedings are often commenced under the section, and abandoned when the inspector of poor undertakes that the lunatic shall be otherwise dealt with; and the Commissioners add that there is no reason to believe that the safety of the public is not secured.

Not fewer than 35 aliens were sent away from Scotland—12 being sent to England and 23 to Ireland—during 1897, and by this step something like £900 a year was saved to the Scottish Exchequer. We have more than once wondered why this proceeding could not be put into force in England, where the saving would amount to hundreds of thousands of pounds; but then they manage these things better in Scotland.

The paragraph relating to the real or supposed increase of lunacy is an exceedingly able and closely reasoned one. We have not space to quote it *in extenso*, but we may assume that the Commissioners do not hold the view that there is an increase, or perhaps they think that no data exist at present for arriving at a conclusion on the point. The paragraph ends as follows: "The figures given show that a greater number of lunatics are, from whatever cause, being sent to asylums; and they also show, as regards private patients, that the increase is confined to persons of middle and advanced age, and as regards pauper patients that, though the numbers admitted have increased at all ages, much the greater proportion of the increase is contributed by persons of middle and advanced age; but it does not appear that they can be safely relied upon as furnishing any larger inference."

The Scottish Commissioners are under the Compulsory Retirement Act, and we believe Dr. Sibbald has reached the age when resignation must be sent in unless extension of service be granted, which has been done in this instance. As a rule we should be opposed to such an extension; but in Dr. Sibbald's case there cannot be a doubt that the Scottish Lunacy Department has gained by the extension, and we heartily congratulate Dr. Sibbald on possessing the vigour which rendered that extension possible.

